# From Severe Virilization to Precocious Puberty in a 46,XX Patient: Therapeutic Challenges in a Case of 11β-Hydroxylase Deficiency

**DOI:** 10.7759/cureus.83965

**Published:** 2025-05-12

**Authors:** Mariam Hamaichat, Asmae Mehdi El Yacoubi, Sanae El Moudden, Sana Abourazzak

**Affiliations:** 1 Endocrinology, Souss Massa University Hospital Centre, Agadir, MAR; 2 Pediatric Endocrinology, Hassan II University Hospital Centre, Fez, MAR

**Keywords:** 11β-hydroxylase deficiency, congenital adrenal hyperplasia, disorders of sex development, precocious puberty, sex assignment

## Abstract

11β-hydroxylase deficiency (11β-OHD) is an autosomal recessive genetic disorder caused by mutations in the *CYP11B1* gene. It most commonly presents with virilization of the external genitalia in both sexes. A delayed diagnosis can be observed in 46,XX patients with severe virilization of the external genitalia who are raised as boys. This report presents the case of a child from a consanguineous marriage who presented at the age of seven with premature pubarche. The diagnosis of congenital adrenal hyperplasia (CAH) due to 11β-OHD was made based on virilization of the external genitalia (Prader stage 5) in a female child (46,XX) raised as a boy, associated with precocious pseudopuberty, and a very high level of 11-deoxycorticosterone. Imaging revealed female-type internal genital organs. The multidisciplinary team recommended feminization surgery; however, this was declined by the parents, and they were subsequently lost to follow-up. At the age of 11, the child presented with true precocious puberty, characterized by breast development (Tanner stage 4) and a pubertal uterus on pelvic ultrasound, accompanied by severe statural and weight delay. A re-evaluation of the therapeutic approach was conducted in consultation with the parents, who subsequently consented to a masculinization procedure to preserve the child’s raised sex. Late diagnosis of CAH poses significant management challenges. This observation highlights the importance of early screening at birth and during medical consultations, as well as the importance of appropriate management to prevent complications related to sex assignment, growth, and fertility and to improve the psychosocial quality of life of the patient and their parents.

## Introduction

11β-hydroxylase deficiency (11β-OHD) is the second most common cause of congenital adrenal hyperplasia (CAH), accounting for 0.2-8% of cases [1]. It is an autosomal recessive genetic disorder caused by mutations in the *CYP11B1* gene, which encodes the enzyme 11β-hydroxylase (11β-OH) [2]. Clinically, 11β-OHD presents with prenatal and postnatal virilization of the external genitalia in both sexes, hyperpigmentation, precocious pseudopuberty, accelerated skeletal maturation, short stature in adulthood, and hyporeninemic hypokalemic hypertension [1]. The absence of salt-wasting syndrome in this deficiency may delay diagnosis in 46,XY patients. Similar diagnostic delay may occur in 46,XX patients with severe virilization of the external genitalia (Prader 5) who are raised as boys [3]. Through this clinical case, we aim to raise awareness of CAH among healthcare professionals, emphasize the importance of early diagnosis and treatment to prevent gender identity issues at an older age, and discuss the management challenges of this condition.

## Case presentation

We report the case of a child from a first-degree consanguineous marriage, the youngest of four siblings. The patient’s family history included a brother who died in the neonatal period (cause unknown) and another sibling who presented with premature pubarche and died at the age of 11. The patient’s medical history began at the age of two, when hypospadias and pubic hair were identified by a nurse during a circumcision examination at a health center. The patient was referred to a pediatrician for further exploration, but the parents did not follow up. At the age of seven, following the death of the elder brother, the parents sought consultation at our facility. A clinical examination was conducted, which revealed a weight at +3 SD, height at +2 SD, and blood pressure of 120/82 mmHg. Examination of the external genitalia revealed a phallus measuring 3.5 cm, exhibiting slight pigmentation and fusion of the labia, a solitary hypospadiac opening, and the absence of palpable gonads. The pubic hair was observed to be at Tanner stage 3. The remainder of the physical examination revealed hyperpigmentation of the hands, feet, and face.

The results of the biological tests revealed a low 8 AM cortisol level of 5.6 µg/dL, an elevated 17-hydroxyprogesterone at 8.6 ng/mL (normal range: <0.89 ng/mL), and an elevated 11-deoxycorticosterone (DOC) at 6,056 pg/mL (normal range: 20-130 pg/mL) (Table 1). The karyotype was determined to be 46,XX, and genetic testing revealed a mutation in the *CYP11B1* gene. The bone age was estimated at 10 years. The patient was initiated on a treatment regimen of hydrocortisone at a dosage of 20 mg/m². The parents were informed of the available treatment options to align the phenotypic sex with the genotypic sex. The legal guardian opted to maintain the male sex, as the child had been raised as a boy. The proposal of masculinization surgery was made; however, the parents were lost to follow-up. This may reflect a combination of psychosocial and emotional challenges often seen in cases of disorders of sex development. Facing complex decisions about gender identity can cause significant psychological distress, social stigma, and cultural pressure, which can lead to disengagement from medical care.

**Table 1:** The key clinical features, hormonal findings, imaging results, and diagnostic criteria at the age of seven.

Parameter	Value
Weight	+3 SD
Height	+2 SD
Pubic hair	Tanner stage 3
Cortisol	5.6 µg/dL (normal range: 5–25 µg/dL)
17-hydroxyprogesterone	8.6 ng/mL (normal range: <0.89 ng/mL)
11-deoxycorticosterone	6,056 pg/mL (normal range: 20–13 pg/mL)
Bone age	10 years
Karyotype	46,XX
Genetic testing	*CYP11B1* mutation

**Figure 1:**
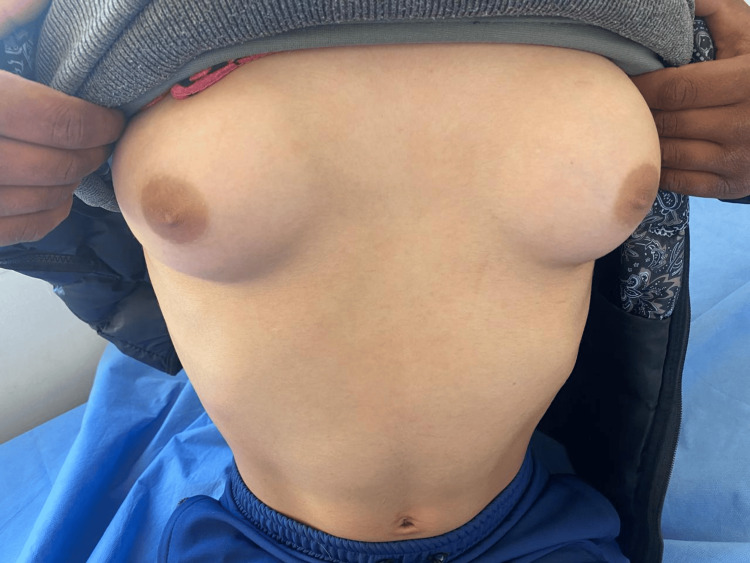
Clinical image showing breast development during central precocious puberty (Tanner stage 4).

**Figure 2:**
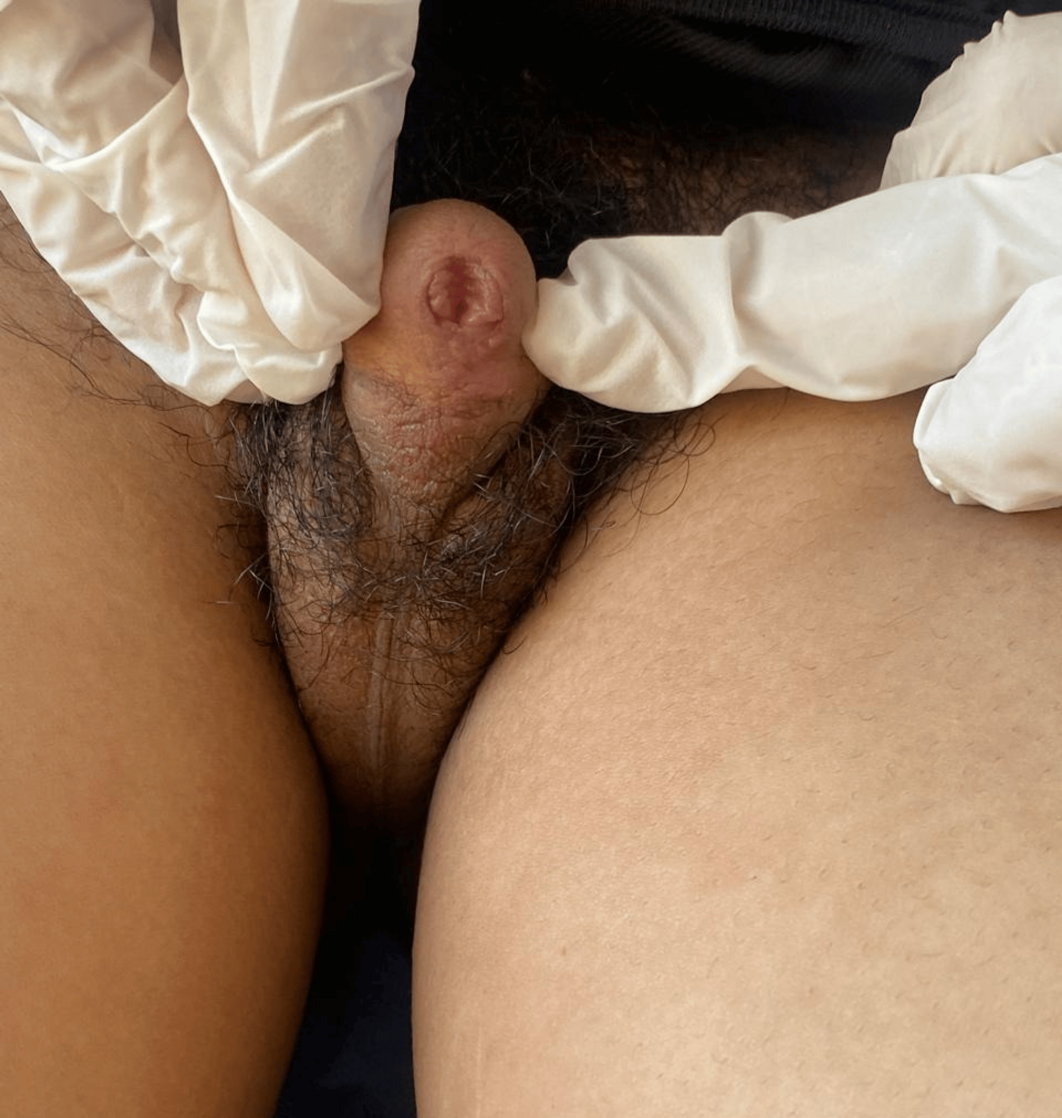
Clinical image showing ambiguous genitalia at Prader stage 5 and pubic hair at Tanner stage 5.

At the age of 11, the patient returned for consultation due to the onset of breast development at Tanner stage 4 (Figure 1). The external genitalia were at Prader stage 5 and pubic hair at Tanner stage 5 (Figure 2). The patient’s weight was at -1.5 SD, while height was at -2 SD. Gonadotropin levels showed follicle-stimulating hormone at 4.48 mIU/mL (normal range: 2-13 mIU/mL), luteinizing hormone at 2.04 mIU/mL (normal range: 1.5-8 mIU/mL), and high estradiol at 52.23 pg/mL (normal range: 34-186 pg/mL). A pelvic ultrasound was performed, revealing a pubertal-sized uterus with a length of 40 mm, a thin and regular endometrium, and a left lateral uterine structure measuring 11 mm in height, resembling the left ovary, with the right ovary not visualized (Table 2). The therapeutic decision was then discussed again with the parents, who consented to masculinization surgery. The child was referred to pediatric surgery for further management. A psychiatric consultation was deemed necessary to evaluate the child’s condition and to support the parents in their decision-making process.

**Table 2:** Clinical examination, hormonal levels, and pubertal staging during follow-up (at the age of 11).

Parameter	Value
Weight	-1.5 SD
Height	-2 SD
Pubic hair	Tanner stage 5
Breast	Tanner stage 4
External genitalia	Prader stage 5
Follicle-stimulating hormone	4.48 mIU/mL (normal range: 2–13 mIU/mL)
Luteinizing hormone	2.04 mIU/mL (normal range: 1.5–8 mIU/mL)
Estradiol	52.23 pg/mL (34–186 pg/mL)
Pelvic ultrasound	Pubertal-sized uterus and a left lateral uterine structure 11 mm in height (left ovary), with the right ovary not visualized

## Discussion

CAH is an autosomal recessive disorder caused by defects in various enzymes responsible for cortisol and aldosterone biosynthesis in the adrenal glands [4]. The condition was first documented in 1865 by De Crecchio during the autopsy of a 46-year-old male who had succumbed to acute adrenal insufficiency [5]. It is estimated that 21-hydroxylase deficiency accounts for over 95% of CAH cases [6]. 11β-OHD is the second most common cause and accounts for 0.2-8% of CAH cases [1]. It has been observed in approximately 1 in 100,000 to 1 in 200,000 live births in populations with low rates of consanguinity, while the prevalence may reach 1 in 60,000 in populations with a high rate of consanguinity [1].

Mutations in the *CYP11B1* gene have been identified as a cause of deficiency in the 11β-OH enzyme. This results in a decreased conversion of 11-deoxycortisol and DOC to cortisol and corticosterone, respectively, in the fasciculata zone of the adrenal cortex [7]. Low cortisol levels activate the negative feedback mechanism of the hypothalamic-pituitary-adrenal axis, resulting in increased adrenocorticotropic hormone production and adrenal cortical hyperplasia [7].

Excessive secretion of DOC and its metabolites, which possess mineralocorticoid activity, has been demonstrated to induce hypertension in 30-66% of cases [1]. In the present case, however, blood pressure remained within the normal range during the follow-up period.

In the case of female patients, the diagnosis is frequently made at birth due to sexual development abnormalities with a 46,XX karyotype. Conversely, in patients with a 46,XY karyotype, diagnosis is frequently delayed and occurs when they present with precocious pseudopuberty, typically before the age of three [8].

Female patients exhibit varying degrees of virilization of the external genitalia, while the internal genitalia are derived from Müllerian structures and the gonads remain intact and functional [7]. This observation was made in the case of the index patient, who exhibited external genitalia at Prader stage 5, accompanied by female-type internal genital organs. The patient’s precocious pseudopuberty progressed into true central precocious puberty, which is responsible for breast development, pubertal-sized uterus, and accelerated growth, leading to a short stature. Indeed, prolonged exposure to androgens has been demonstrated to alter the control of the gonadotropic axis, leading to the onset of true central precocious puberty after the initiation of treatment with glucocorticoids, which attenuates the production of adrenal androgens and, therefore, lifts their inhibition on the gonadotropic axis [8].

Impaired final height has been observed in patients diagnosed with 11β-OHD, regardless of their age at the time of diagnosis or the extent of clinical and biochemical control. The mean final height of females is 10 cm below that of the general population and 18 cm below that of males who reach the age of 18 [7]. This short stature is attributable to accelerated skeletal maturation and premature epiphyseal closure, a consequence of excess androgens. Glucocorticoid treatment has also been observed to contribute to reduced final height by decreasing endogenous growth hormone (GH) secretion and insulin-like growth factor 1 bioactivity [9].

Genotype-phenotype correlation has been documented in 11β-OHD. To date, more than 200 mutations, predominantly point mutations, have been identified [1]. Founder mutations in the *CYP11B1* gene have been documented in various ethnic groups, including the p.R448H mutation observed in Moroccan Jews [10], the p.N394Rfs*37 and p.L299P mutations noted in Turkish individuals [11], and the p.Q356X and p.G379V mutations seen in Tunisians [12].

The indication for feminizing genitoplasty appears to be substantiated in 46,XX patients diagnosed with 11β-OHD. The most commonly recommended surgical method is early clitorovaginoplasty, which is performed in a single step [13]. According to the Endocrine Society’s recommendations, it is better to observe or delay surgery in less virilized girls, but early reconstruction is recommended in severely virilized girls with CAH [14].

When the diagnosis is delayed, as was the case in this patient, the decision-making process becomes more complex. It can be challenging for both the parents and the patient to accept a change in raised sex. Many studies have shown a high prevalence of psychiatric disorders among individuals with disorders of sex development. A study conducted in the United States found that 53.6% of people with disorders of sex development (n = 198) rated their mental health as fair or poor, compared with 17.7% of the general population. Additionally, 40.9% of people with disorders of sex development reported post-traumatic stress disorder, and 31.8% had a history of suicide attempts [15].

Hithayathulla et al. [16] reported a similar case of CAH due to 11β-OHD in a patient raised as male who presented at the age of 20 years with intermittent hematuria and breast development, the karyotype was 46,XX and the internal genitalia were female. Psychiatric assessment revealed that the patient preferred a male gender identity and gender reassignment surgery was performed.

In our case, the disadvantages of changing the phenotypic sex compared to the genotypic sex, surgical interventions, fertility issues, and sexual functioning were explained to the patient’s legal guardians, who opted to maintain the male sex. The patient was referred for surgery involving a total hysterectomy, bilateral gonadectomy, and mastectomy. It is recommended that patients with sexual development disorders and their parents undergo a psychiatric consultation and assessment [13].

## Conclusions

11β-OHD is a rare genetic disorder. Diagnosis and management present several challenges. Phenotypic variability and the rarity of the condition are the primary reasons for delayed diagnosis. The case presented in this report illustrates the medical and ethical complexities of managing 46,XX patients with 11β-OHD who are raised as boys. Early management of these patients is crucial to prevent long-term complications, particularly in terms of gender identity, growth, and fertility. The ethical dilemmas, especially regarding sex assignment, highlight the importance of psychiatric support for parents and patients. Therefore, there is a need for increased awareness among healthcare professionals to improve screening and management of these rare diseases.
